# Hypoperfusion With Vomiting, Abdominal Pain, or Dizziness and Convulsions: An Alert to Fulminant Myocarditis in Children

**DOI:** 10.3389/fped.2020.00186

**Published:** 2020-05-05

**Authors:** Angang Zhu, Tian Zhang, Xiaobi Hang, Xiaoguang Zhang, Yingying Xiong, Tao Fang, Mingwu Chen

**Affiliations:** ^1^Department of Pediatrics, Anhui Provincial Hospital, Wannan Medical College, Hefei, China; ^2^Department of Pediatrics, Anhui Provincial Hospital Affiliated to Anhui Medical University, Hefei, China; ^3^Department of Pediatrics, Anhui Provincial Children's Hospital, Hefei, China; ^4^Division of Life Sciences and Medicine, Department of Pediatrics, The First Affiliated Hospital of USTC, Anhui Provincial Hospital, University of Science and Technology of China, Hefei, China

**Keywords:** hypoperfusion, fulminant, myocarditis, children, retrospective analysis

## Abstract

**Objective:** To investigate the clinical features, treatment methods, and outcomes of fulminant myocarditis (FM) in children.

**Methods:** The clinical data of 23 children with FM hospitalized in the First Affiliated Hospital of USTC, Division of Life Sciences and Medicine, University of Science and Technology of China (Anhui Provincial Hospital) and Anhui Provincial Children's Hospital from January 2011 to September 2019 were retrospectively analyzed.

**Results:** Among the 23 patients analyzed, 10 were male and 13 were female. The patients aged from 6 months to 14 years old (6.5 ± 3.4 years), and 18 patients were over 3 years old. There were 14 cases with respiratory symptoms, 16 cases with gastrointestinal symptoms, 15 cases with neurological symptoms, and 19 cases with hypoperfusion manifestations. Creatine kinase MB (CK-MB) and cardiac troponin I (CTnI) levels were increased in 19 and 21 cases, respectively. Electrocardiography (ECG) showed ST-T changes in 18 cases and atrioventricular blocks (AVB) in 15 cases. Echocardiography (ECHO) showed cardiac chamber enlargement (CCE) in eight cases, left ventricular systolic dysfunction in five cases, decrease in left ventricular ejection fraction (LVEF) in four cases, reduction in wall motion in two cases, and pericardial effusion in seven cases. Intravenous immunoglobulin (IVIG) and glucocorticoids were administered to 19 and 20 patients, respectively. Fourteen patients were treated with temporary pacemakers, one patient received extracorporeal membrane oxygenation (ECMO), one patient received continuous renal replacement therapy (CRRT), and one patient received ECMO combined with CRRT. Twenty patients improved at discharge, and three patients died.

**Conclusion:** Preschool and school-age children showing hypoperfusion symptoms, such as paleness, cold, clammy limbs, and capillary refill time (CRT) extension, accompanied by vomiting, abdominal pain, dizziness, convulsions, and other symptoms, should be carefully examined for FM. CK-MB, CTnI, ECG, and echocardiogram need to be performed at the earliest opportunity. In the early stages of FM, vital signs should be actively monitored, high-dose IVIG and glucocorticoids should be administered, and life support technologies such as temporary pacemakers, ECMO, and CRRT should be used to increase the survival rate of children with FM as needed.

## Introduction

Fulminant myocarditis (FM) is an inflammatory process of the myocardium that is an important cause of cardiac dysfunction in children and is characterized by abrupt onset, fast progress, and high mortality (1, 2). Patients may present with acute heart failure, cardiogenic shock, Adams-Stokes syndrome, or fatal arrhythmia in a short time and are usually admitted to the hospital with digestive system symptoms such as vomiting and abdominal pain or neurological symptoms such as dizziness and convulsions (3). The initial clinical symptoms are often atypical and can easily be misdiagnosed. The aim of this study was to improve our understanding of the diagnosis and treatment of FM by analyzing the clinical features, treatment methods, and outcomes in children with FM.

## Materials and Methods

### Research Subjects

Data from 23 children with a diagnosis of FM hospitalized in the First Affiliated Hospital of USTC, Division of Life Sciences and Medicine, University of Science and Technology of China (Anhui Provincial Hospital) and Anhui Provincial Children's Hospital from January 2011 to September 2019 were retrospectively analyzed.

### Ethics Statements

This study was approved by the ethics committee of the First Affiliated Hospital of USTC, Division of Life Sciences and Medicine, University of Science and Technology of China (Anhui Provincial Hospital) and Anhui Provincial Children's Hospital, and written informed consent was obtained from the parents of the study participants.

#### Inclusion Criteria

All selected children were diagnosed with FM and were younger than 16 years old. The diagnosis of FM was based on clinical manifestations, electrocardiography (ECG), and echocardiography, which is in line with the criteria for the clinical diagnosis of myocarditis in the Diagnostic Recommendations for Children with Myocarditis (2018 edition) (4) and the diagnostic criteria for FM recommended by Ammirati et al. (5). The following clinical manifestations were considered for the diagnosis of FM: acute onset, cardiac hemodynamic instability, hemodynamic or circulatory support to maintain heart function or blood pressure, and evidence of myocardial damage suggesting cardiac dysfunction, such as changes in CK-MB levels, CTnI levels, ECG, and echocardiography.

#### Exclusion Criteria

Congenital heart disease, non-ischemic cardiomyopathy, endocardial elastic fibrosis, and myocardial infarction.

### Research Methods

The following clinical data of the 23 children were reviewed: age; gender; clinical manifestations; myocardial injury biomarkers, such as CK-MB, CTnI, N-terminal pro-B-type natriuretic peptide (NT-pro-BNP), and B-type natriuretic peptide (BNP) levels; ECG; echocardiography; treatment methods; outcomes.

### Clinical Treatment

All 23 children received treatments including bed rest, oxygen, anti-infective therapy, myocardial nutrition, anti-shock treatment, anti-heart failure treatment, anti-arrhythmia treatment, and other comprehensive treatments after admission. IVIG, glucocorticoids, temporary pacemakers, ECMO, and CRRT were administered according to the condition of the patients.

### Statistical Analysis

The SPSS 21.0 statistical software was used for statistical analysis. The measured data are expressed as mean ± standard deviation, and the count data are expressed as percentages (%).

## Results

The main clinical data of the 23 children with FM are shown in Table 1.

**Table 1:** Main clinical data of 23 children with FM.

**Patient**	**Age**	**Gender**	**Symptoms**	**CK-MB (IU/L)**	**CTnI (μg/L)**	**ECG**	**ECHO**		**Treatment**	**Outcome**
							**Changes**	**LVEF (%)**		
1	8 years	M	H, NSS, DSS	72	20.069	ST-T change, AVB	/	67	IVIG, GC, TP	Improve
2	7 years	F	H, NSS, DSS, RSS	129	>50	AVB	PE, RIWM	48	IVIG, TP, CRRT	Died
3	35 months	F	DSS, RSS	118	38.13	ST-T change, AVB	/	53	IVIG, GC, TP	Died
4	8 years	F	H, NSS, DSS	95	8.762	ST-T change, AVB	PE	53	IVIG, GC, TP	Improved
5	11 years	F	H, NSS, DSS, RSS	11	23.476	ST-T change, AVB	PE	61	IVIG, GC, TP	Improved
6	7 years	M	H, NSS, DSS, RSS	806	4.67	ST-T change, AVB	PE	58	IVIG, GC, TP	Improved
7	32 months	F	H, DSS, RSS	24	0.03	ST-T change, AVB	/	73	IVIG, GC, TP	Improved
8	11 years	M	/	47	15.955	ST-T change, AVB	/	62	IVIG, GC, TP	Improved
9	9 years	F	H	39	8.51	ST-T change	RIWM	36	IVIG, GC	Improved
10	8 years	F	DSS	67	18.296	ST-T change	/	75	IVIG, GC	Improved
11	8 years	F	H	30	3.089	ST-T change	/	62	/	Improved
12	10 years	M	H, NSS, RSS	8.3	0.06	ST-T change, AVB	CCE	66	GC, TP	Improved
13	6 months	F	DSS, RSS	20	0.017	AVB	/	66	IVIG, GC	Improved
14	8 years	F	H, NSS, DSS, RSS	82	52.2	ST-T change, AVB	/	65	IVIG, GC, TP	Improved
15	14 years	M	H, NSS, RSS	116.5	2.9	/	CCE	72	IVIG, GC	Improved
16	5 years	M	H, NSS, RSS	39.83	3.4	ST-T change, AVB	LVSD, CCE	67	IVIG, GC, TP	Improved
17	1 years	M	H, NSS	46.4	6.25	ST-T change	/	/	/	Died
18	4 years	M	H, DSS	26.5	13.3	ST-T change	LVSD, PE, CCE	53	IVIG, GC	Improved
19	3 years	F	H, NSS, DSS, RSS	56.3	8.7	ST-T change, AVB	/	59	IVIG, GC, TP	Improved
20	7 years	F	H, NSS, DSS, RSS	156.19	29.9	ST-T change, AVB	LVSD, PE, CCE	58	IVIG, GC, TP, ECMO	Improved
21	1 years	F	H, NSS, DSS, RSS	11.49	1.84	ST-T change	LVSD, CCE	22	IVIG, GC, ECMO, CRRT	Improved
22	10 years	M	H, NSS, DSS	15.08	0.993	AVB	CCE	64	GC, TP	Improved
23	5 years	M	H, NSS, DSS, RSS	22	1.77	/	LVSD, PE, CCE	26	IVIG, GC	Improved

*CK-MB, creatine kinase MB; CTnI, cardiac troponin I; ECG, electrocardiography; ECHO, echocardiography; LVEF, left ventricular ejection fraction; H, hypoperfusion; NSS, neurological system symptoms; DSS, digestive system symptoms; RSS, respiratory system symptoms; AVB, atrioventricular block; PE, pericardial effusion; RIWM, reduction in wall motion; CCE, cardiac chamber enlargement; LVSD, left ventricular systolic dysfunction; IVIG, intravenous immunoglobulin; GC, glucocorticoid; TP, temporary pacemaker; CRRT, continuous renal replacement therapy; ECMO, extracorporeal membrane oxygenation*.

### Age and Gender

**Figure 1:**
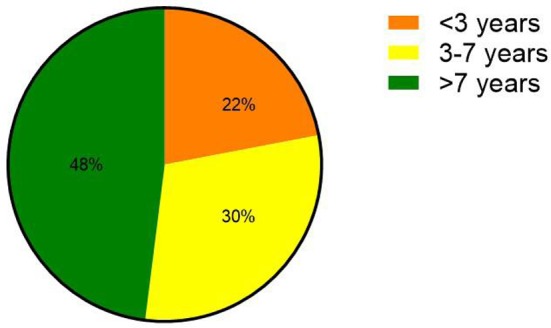
Age distribution of children with FM.

Among the 23 patients analyzed, 10 were male and 13 were female. The patients were aged from 6 months to 14 years old (6.5 ± 3.4 years). Five patients (22%) were under the age of 3. Eighteen patients (78%) were over 3 years old, of which seven patients (30%) were aged between 3 and 7 years old, and 11 patients (48%) were aged over 7 years old (Figure 1).

### Clinical Manifestations

**Figure 2:**
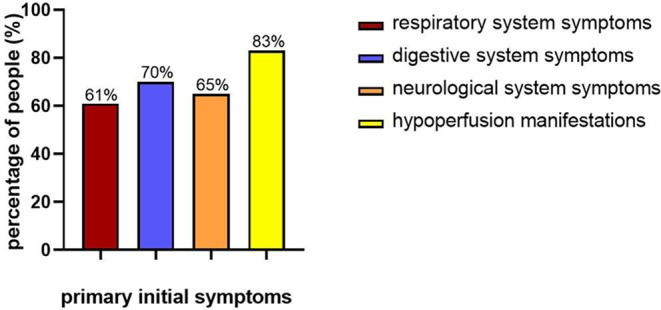
Primary initial symptoms of children with FM.

The initial symptoms of children with FM were varied. Respiratory symptoms such as fever and cough occurred in 14 patients (61%); digestive system symptoms such as nausea, vomiting, and abdominal pain occurred in 16 patients (70%); 15 patients (65%) presented with neurological symptoms such as headache, dizziness, syncope, convulsion, drowsiness, and coma. Regarding the circulatory symptoms, there were 19 patients (83%) with hypoperfusion manifestations, such as paleness, cold, clammy limbs, and capillary refill time (CRT) extension (>3 s), and 10 patients (43%) with chest pain, chest tightness, and palpitation (Figure 2). The time from onset to admission was 0–5 days (2.4 ± 1.3 days). Fourteen patients (61%) suffered from low blood pressure, eight patients (35%) had heart failure, and five patients (22%) had hepatosplenomegaly. One child was admitted to the hospital due to fever and convulsions, accompanied by pale complexion and hypotension, and then quickly developed shock and heart failure, and finally died after 2 h.

### Myocardial Injury Biomarkers

Twenty-three children were admitted to the hospital for testing myocardial injury biomarkers. CK-MB levels (normal physiological range: 0–16 IU/L) were increased in 19 children (83%), which peaked at 1–8 days (3.4 ± 2.3 days) after onset and returned to the normal range in 14 patients after 3–17 days (8.7 ± 4.3 days). CTnI levels (normal physiological range: 0–0.03 g/L) were increased in 21 cases (91%), peaking at 1–6 days (3.0 ± 1.6 days) after onset and returning to the normal range in 19 patients after 7–29 days (14.4 ± 6.7 days). An NT-pro-BNP examination was completed for 10 patients, and the resulting value was high in eight patients (>300 pg/mL). BNP examination results are available for seven patients, and the value was high in six patients (>100 pg/mL).

### ECG

**Figure 3:**
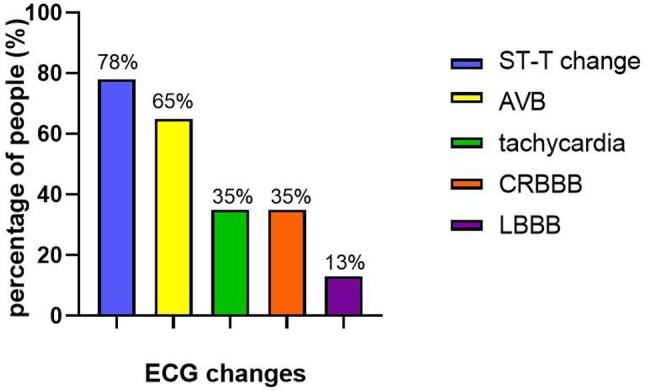
ECG changes of children with FM.

All children were examined using a standard 12-lead ECG after admission. ST-T abnormalities were found in 18 children (78%). There were 15 cases (65%) with atrioventricular blocks (AVB), of which three cases (13%) were second-degree AVB and 12 cases (52%) were third-degree AVB. The AVB occurred between days 1 and 6 (3.6 ± 1.6 days) after onset, and the patients recovered after 1–14 days (6.9 ± 4.3 days). There were eight cases (35%) with tachycardia, most of which were sinus tachycardia cases. The manifestations of the child who died quickly were ST-T changes and sinus tachycardia, quickly progressing into cardiac arrest. Complete right bundle branch block (CRBBB) occurred in eight children (35%), and left bundle branch block (LBBB) occurred in three children (13%). At the time of discharge, there were six children with CRBBB and two children with LBBB (Figure 3).

### Echocardiography

Twenty-two children underwent echocardiography examination (the child who died quickly did not undergo this examination). Cardiac chamber enlargement (CCE) occurred in eight cases (36%): there were three cases of left cardiac enlargement and five cases of total cardiac enlargement. Four patients with CCE recovered after 12–19 days (14.8 ± 2.9 days), and the other patients did not recover, even at discharge. Five cases (23%) presented with left ventricular systolic dysfunction: two recovered within 10 days, one recovered after 19 days, and two did not recover even at discharge. Four cases (18%) had a decreased left ventricular ejection fraction (LVEF) (<50%), of which it was restored in two cases (one died, and one did not recover). Two cases (9%) had a reduction in wall motion, and seven cases (32%) had mild pericardial effusion.

### Treatment and Outcome

All patients were treated with conventional treatments, including bed rest, oxygen, anti-infective therapy, myocardial nutrition, anti-shock, anti-heart failure, and anti-arrhythmia treatments. IVIG (total 2 g/kg over a period of 2 days) was used in 19 patients (83%). Twenty patients (87%) were treated with glucocorticoid therapy (methylprednisolone 10 mg/kg/d for 3 days and prednisolone 2 mg/kg/d, decreased gradually and maintained for 18–24 weeks based on the condition). IVIG combined with glucocorticoids was administered to 18 patients (78%). The CTnI levels decreased after treatment with IVIG and/or glucocorticoids in 19 patients. Of the 15 patients with second-degree AVB or third-degree AVB, 14 (61%) received temporary pacemakers for 2–24 days (9.3 ± 6.6 days), and the remaining one patient rejected pacemaker implantation. For the patients with cardiogenic shock after conventional therapy, the following treatments were used: ECMO was used in one patient for 7 days, CRRT was used in one patient for 8 h, ECMO combined with CRRT was used in one patient for 5 days, and the child who was only treated with CRRT developed multiple organ failure leading to death. Overall, 20 (87%) of the 23 patients improved, and three patients (13%) died. Clinical symptom recovery was achieved in the children who survived, and most examination indicators were gradually restored to the normal range. ECG was assessed after 6 months of follow-up in nine children, of which six were cured and two presented with CRBBB, and the status of one was unknown (the cellphone had no service).

## Discussion

Myocarditis is usually related to viral infections and post-viral immune-mediated responses. FM is the most severe type of viral myocarditis. It has been reported that the mechanisms of this disease progression are associated with an immunoreaction. Viral infection results in damage to myocardial cells, which can induce immune responses causing cardiac damage. Necrotic myocardial cells may release autoantigens, which can activate the immune reaction leading to the obstruction of nerve-body fluid regulation, myocardial remodeling, and myocardial dysfunction. In more serious instances, it can cause heart failure, cardiogenic shock, and sudden death (1). FM can occur in all age groups of children. In this study, 78% of children with FM were over 3 years old, showing that the majority were preschool and school-age children. At the onset, 70% of patients had digestive system symptoms, and 65% had neurological symptoms. The non-cardiac manifestations mainly included digestive system symptoms, such as vomiting and abdominal pain or neurological system symptoms such as dizziness and convulsions, which was consistent with the results reported in other studies (6). FM can cause extensive or localized necrosis of myocardial cells or tissues, severe heart pumping dysfunction, and decreased cardiac output, leading to a decrease in effective blood volume (1, 7), which results in a series of hypoperfusion manifestations of organs and tissues. The inadequacy of effective circulatory blood volume can start the compensatory mechanism of the body and activate the sympathetic-adrenal medulla system, subsequently raising the level of catecholamines. This hormone can cause cutaneous vasoconstriction and reduce cutaneous blood flow, leading to a pale complexion. At the same time, the secretion of sweat glands is increased, and the skin becomes wet, which manifests as cold, clammy limbs. Low blood volume affects the recovery of capillary blood flow after being pressed (8). Around 83% of children showed paleness, cold, clammy limbs, and CRT extension, which were considered to be connected with heart-pumping function dysfunction due to FM. These clinical manifestations, which are often ignored at the first visit, should be paid more attention.

Biomarkers of myocardial injury are widely used in the clinical diagnosis of heart diseases. Creatine kinase (CK) and CTnI are common markers for detecting myocardial injury. Wang et al. reported that the rise of CK is an early feature of FM (9). Earlier studies found that the levels of CK-MB and CTnI in patients with FM were elevated more significantly than in patients without FM and that CTnI values were more sensitive and specific (10). In addition, recent studies have shown that NT-Pro-BNP and BNP levels can reflect impaired cardiac function, which is conducive to the early identification of FM. Levels of CTnI >1 μg/L and high BNP levels are risk factors that influence the prognosis of patients (3, 11). In this study, the biomarkers of myocardial injury were increased diversely in most children in the early stages of FM, which is useful since it is suggested that early detection is helpful for the diagnosis of this disease. After treatment, these indicators gradually returned to the normal range, showing that it can be used to judge recovery and prognosis.

ECG and echocardiography are important techniques for the diagnosis of FM. Electrocardiography of patients with FM was characterized by ST-T changes, AVB, and various ectopic arrhythmias in the acute phase (12); 65% of children with FM presented ST-T changes with AVB, and second-degree AVB was more common. The main echocardiography findings in children with FM were decreased ventricular wall motion and ejection fraction, thickened ventricular wall, heart dilation, pericardial effusion, valvular regurgitation, and endoluminal thrombosis (13). The decrease in ejection fraction indicates cardiac insufficiency, and the reduction of ventricular wall motion amplitude and ejection fraction can reflect the severity of FM, which are important indicators for evaluating the prognosis of children with FM (14). Decreased LVEF, pericardial effusion, and decreased ventricular wall motion amplitude occurred in a small number of patients in this study. The small number of samples or the non-acute phase of the disease at the time of testing may be the reason for the low positivity rate of echocardiography. Endomembrane biopsy (EMB) is important for diagnosing FM, but it is invasive (15). In the current practice, EMB is seldom used to diagnose myocarditis because of the unstable hemodynamics in patients and the risks inherent to the procedure. In addition, cardiac magnetic resonance (CMR) is a valuable non-invasive method that can be applied for the diagnosis of FM with high sensitivity and specificity (16). Myocardial perfusion imaging is also helpful (17).

Of the 19 patients with hypoperfusion, 15 showed increased CK-MB levels, 17 showed increased CTnI levels, 17 had positive findings on ECG, and 13 had positive results on echocardiography for FM, suggesting that the manifestations of hypoperfusion have implications for the diagnosis of FM. The positivity rate of various checks in patients who had neurological symptoms or gastrointestinal symptoms was similar to that in patients with hypoperfusion. Therefore, we recommend that patients who present hypoperfusion accompanied by neurological symptoms or digestive system symptoms should be alerted to the possibility of FM.

Early comprehensive treatment of FM is essential. Treatment with bed rest, oxygen, anti-infective therapy, myocardial nutrition, anti-shock, anti-heart failure, and anti-arrhythmia treatments should be used to maintain heart function. One study has indicated that the early use of glucocorticoids in large doses is effective in the treatment of FM and can reduce mortality (18). Chen et al. found that glucocorticoids may improve cardiac function but cannot reduce mortality (19). Studies have shown that IVIG is beneficial for the treatment of FM and can improve LVEF and long-term prognosis of patients with this disease (20, 21). However, the use of IVIG and glucocorticoids as immunotherapy for this condition remains controversial. Around 78% of patients received an early application of IVIG combined with glucocorticoids, leading to only one child death (of the three children who died, one died soon after admission without the application of glucocorticoids and IVIG, and glucocorticoids alone were used for the other), suggesting that early immunotherapy can effectively improve the success rate of rescue. In recent years, temporary pacemakers, ECMO, CRRT, and other life support technologies have been gradually applied to FM. Patients can manifest Adams-stokes syndrome in a short period of time and even suffer cardiac shock and cardiac arrest. The installation of a temporary pacemaker is a quick and effective treatment measure to save lives. The timing of temporary pacemaker implantation directly determines the treatment effect and prognosis (22). Fourteen patients with AVB received temporary pacemakers to maintain stable hemodynamics, of which only one patient died. Lorusso et al. pointed out that ECMO could provide strong circulatory support for patients with FM and cardiogenic shock on the basis of 5 years of multi-institutional experience (23). A weighted meta-analysis of 170 patients with FM indicated that the survival rate at discharge was nearly 66.9% after ECMO treatment (24). For patients who are not hemodynamically stable with conventional treatments or even pacemakers, ECMO combined with CRRT may be a solution to the problem (25). One patient who was treated with ECMO and one patient who received ECMO combined with CRRT were alive at discharge. However, one patient with hemodynamic instability only accepted CRRT and soon died of multiple organ failure. Our data suggest that immunotherapy is important for treating FM; temporary pacemakers should be used in patients with AVB, and ECMO combined with CRRT should be applied to patients with circulation instabilities after conventional treatments.

There are some limitations to our study. This is a small-sized retrospective clinical study. The conclusions of this study still need to be proven in more multicenter and large-scale clinical studies. Due to technical and financial issues, CMR or myocardial perfusion imaging could not be performed.

## Conclusion

The onset of FM is rapid, and the clinical manifestations vary, which can quickly lead to the death of children with cardiogenic shock. Therefore, early and accurate diagnosis is essential. When preschool and school-age children present with hypoperfusion manifestations such as paleness, cold, clammy limbs, and CRT extension, accompanied by vomiting, abdominal pain, dizziness, convulsions, and other such symptoms, they should be carefully examined for FM. Timely measurements of CK-MB and CTnI levels, together with ECG and echocardiogram, are essential. Reasonable treatment is of great significance in improving the prognosis of children with FM. In the early stage of FM, vital signs should be actively monitored, high-dose IVIG and glucocorticoids should be administered, and life support technologies such as temporary pacemakers, ECMO, and CRRT should be used to increase the survival rate of children with FM as and when needed.

## Data Availability Statement

All datasets generated for this study are included in the article/supplementary material.

## Ethics Statement

The studies involving human participants were reviewed and approved by the Ethics Committee of the First Affiliated Hospital of USTC, Division of Life Sciences and Medicine, University of Science and Technology of China (Anhui Provincial Hospital) and Anhui Provincial Children's Hospital. Written informed consent to participate in this study was provided by the participants' legal guardian/next of kin.

## Author Contributions

AZ has obtained the approval of all other co-authors to submit this article. All the authors have contributed to the manuscript.

## Conflict of Interest

The authors declare that the research was conducted in the absence of any commercial or financial relationships that could be construed as a potential conflict of interest.
